# Different populations of Wnt-containing vesicles are individually released from polarized epithelial cells

**DOI:** 10.1038/srep35562

**Published:** 2016-10-21

**Authors:** Qiuhong Chen, Ritsuko Takada, Chiyo Noda, Satoru Kobayashi, Shinji Takada

**Affiliations:** 1 Okazaki Institute for Integrative Bioscience, National Institutes of Natural Sciences, Okazaki, Aichi 444-8787, Japan; 2 National Institute for Basic Biology, National Institutes of Natural Sciences, Okazaki, Aichi 444-8787, Japan; 3 The Graduate University for Advanced Studies (SOKENDAI), Okazaki, Aichi 444-8787, Japan; 4 Life Science Center, Tsukuba Advanced Research Alliance, University of Tsukuba, Tsukuba, Ibaraki 305-8577, Japan

## Abstract

Accumulating evidence suggests that exosomes are heterogeneous in molecular composition and physical properties. Here we examined whether epithelial cells secrete a heterogeneous population of exosomes, and if that is the case, whether epithelial cell polarity affects release of different populations of exosomes, especially that of those carrying Wnt. Sucrose-density ultracentrifugation and molecular marker analysis revealed that different populations of exosomes or exosome-like vesicles were released from MDCK cells depending on the cell polarity. Wnt3a associated with these vesicles were detectable in culture media collected from both apical and basolateral sides of the cells. Basolaterally secreted Wnt3a were co-fractionated with a typical exosomal protein TSG101 in fractions having typical exosome densities. In contrast, most of apically secreted Wnt3a, as well as Wnt11, were co-fractionated with CD63 and Hsp70, which are also common to the most exosomes, but recovered in higher density fractions. Wnt3a exhibiting similar floatation behavior to the apically secreted ones were also detectable in the culture media of Wnt3a-expressing L and HEK293 cells. The lipidation of Wnt3a was required for its basolateral secretion in exosomes but was dispensable for the apical one. Thus, epithelial cells release Wnt via distinct populations of vesicles differing in secretion polarity and lipidation dependency.

In intercellular communications, signal proteins are transmitted via several different ways, one of which is mediated by extracellular vesicles (EVs). Cells release different types of EVs, including exosomes, microvesicles, and apoptotic bodies, which are distinguished by their biogenetic pathways^1^,^2^,^3^,^4^. Exosomes, which are nanomembranous vesicles ranging in size from 40 to 100 nm in diameter, are originally formed within multivesicular bodies (MVBs) as intraluminal vesicles (ILVs) by inward budding of endosomes and are released upon fusion of MVBs with the plasma membrane^5^. Most exosomes are rich in characteristic proteins, including tetraspanins CD9, CD63, and CD81, as well as heat-shock proteins such as Hsp70^6^. In addition, exosomes are enriched in components involved in the biogenesis of MVBs such as ESCRT (the endosomal sorting complex required for transport)-related proteins, including tumor susceptibility gene 101 protein (Tsg101)^6^,^7^. Exosomes also contain some specific cytoplasmic proteins such as actin, annexins, and glyceraldehyde-3-phosphate dehydrogenase and membrane microdomain proteins including flotillins^6^.

Accumulating evidence suggests that exosomes appear to be heterogeneous in terms of their molecular and physical characteristics. Several studies indicated that cells secrete different populations of exosomes, which can be separated by sucrose density-gradient ultracentrifugation^8^,^9^,^10^,^11^. Other evidence also indicates that different exosome populations are enriched in distinct exosome markers that are secreted from many types of cells^6^. The formation of such different populations of exosomes appears to be distinctly regulated. For instance, the release of exosomes bearing CD63 and Hsp70 is dependent on syndecans or their cytoplasmic adaptor syntenin, but the release of flotillin-positive exosomes is not^12^. In addition, potential heterogeneity in exosome formation inside MVBs is also suggested. While the ESCRT machinery is a well characterized sorting mechanism involved in ILV formation in MVBs, an ESCRT-independent mechanism has also been described^13^,^14^,^15^. These findings raise the question as to whether the heterogeneity of exosomes is relevant to the regulation of intercellular communications via exosomes.

Epithelial cells are one of the cell types that release exosomes. In polarized epithelial cells, intracellular membrane traffic, including MVB delivery to the plasma membrane, is asymmetric. Probably related to this asymmetry, the polarized delivery of exosomes has been described in several cases^16^,^17^,^18^. For instance, αβ crystallin-carrying exosomes are secreted from the apical side of retinal pigment cells^18^. Interestingly, proteome analysis of 2 distinct populations of exosomes released from cells of a human colon carcinoma cell line revealed that molecules trafficking to the apical or basolateral side are differently enriched in these 2 exosome populations, suggesting that epithelial cells release different populations of exosomes from their apical and basolateral surfaces^19^. Thus, some cargos appear to be selectively sorted into different exosomes depending on epithelial polarity, although the precise mechanism underlying polarity-dependent cargo secretion via exosomes remains unclear.

Wnt proteins, which mediate intercellular signaling during embryogenesis and homeostasis^20^,^21^,^22^, are known to be cargos packaged in exosomes^23^,^24^,^25^,^26^,^27^; although it is still debatable whether exosomes play a role as the major conveyors of Wnt proteins. The epithelial cell is one of the sources of Wnt production in developing and adult tissues^28^,^29^. In epithelial cells, Wnt appears to be synthesized and secreted in a polarity-dependent manner. For instance, in Drosophila embryos, the mRNA and proteins of Wingless (Wg), the main Drosophila Wnt, accumulate in the apical region of epithelial cells^29^,^30^,^31^,^32^. In contrast to this cytoplasmic localization, Wg is extracellularly concentrated on the basolateral surface of its producing cells in imaginal discs^29^,^32^. A recent study revealed that Wg is presented first on the apical surface of these cells and then is transferred to the basolateral one via transcytosis^33^. On the other hand, experiments with cultured epithelial cells indicated that mouse Wnt11 secretion mainly occurs at the apical side and that Wnt3a is preferentially secreted from the basolateral side^34^. Given that extracellular movement is not identical between apically and basolaterally secreted Wnt proteins, it appears an important issue to reveal the molecular basis involved in a polarity-dependent secretion of Wnt.

Since the exosome-mediated release of proteins is likely to be dependent on the epithelial polarity, it seems plausible to speculate that exosome-mediated transportation is a candidate system involved in a polarity-dependent secretion of Wnt proteins. Interestingly, flotillin2 (Flo2; also referred to as reggie1), which is abundant in caveolae/lipid rafts and exosomes, stimulates the apical secretion of Wg. Upon overexpression of Flo2, Wg diffusion is increased in the apical surface of the epithelial sheet of the wing disc; whereas knockdown of reggie1 affects the Wg signaling at the apical side^35^. Since Flo2 is involved in the long-range activation of Wg signaling, it seems possible that exosome-mediated Wnt transport is involved in the polarized secretion and possibly in regulation of the signaling range of Wnt proteins^35^,^36^. However, it still remains to be elucidated whether Wnt-associated exosomes are secreted in an apico-basal polarity-dependent manner and, if this is the case, how its polarized distribution is regulated in the biogenesis of exosomes. To answer these questions, we examined exosome-mediated secretion of Wnt proteins by utilizing Madin-Darby canine kidney (MDCK) cells, which can generate a properly polarized epithelial sheet in culture^37^. We found that heterogeneous populations of exosome-like extracellular vesicles were secreted in a polarity-dependent manner and that the secreted Wnt proteins were differently packaged into distinct populations of vesicles depending on the cell polarity.

## Results

### Heterogeneity of exosomes secreted from MDCK cells

To characterize Wnt proteins secreted from polarized epithelial cells, we established MDCK type II cells stably expressing mouse Wnt3a (MDCK-3a). Parental MDCK and MDCK-3a cells were cultured to form a polarized monolayer on a filter support^37^, which can separate the apical and basolateral compartments, as shown in Supplemental Fig. S1a. After confirmation of epithelial sheet formation by monitoring the transepithelial resistance value, we cultured the cells for 24 hours, with replacement of the medium with the same amount of fresh medium at both the apical and basolateral sides. Equal amounts of conditioned medium from the apical and basolateral sides were collected, and the amounts of secreted Wnt3a were examined by Western blotting. As previously reported, the majority of Wnt3a was secreted basolaterally in polarized MDCK-3a cells, whereas a lesser amount of it could also be detected in medium collected from the apical side (Supplemental Fig. S1b,c)^34^.

By using this system, we examined whether Wnt3a secreted from polarized epithelial cells was associated with exosomes. Conditioned medium from either the apical or basolateral side of MDCK and MDCK-3a cells was collected after 24 h of incubation as stated above and then subjected to ultracentrifugation to pellet the exosomes according to a standard exosome-enrichment protocol^38^. Specifically, the conditioned medium was centrifuged at 2000 × g to remove dead cells, followed by centrifugation at 10,000 × g to eliminate cell debris and any large-sized vesicles such as apoptosis vesicles. The collected supernatants were referred to as the S10 sup, which was then ultracentrifuged at 100,000 × g to pellet the small-sized vesicles. Since it was earlier shown that exosomes are abundant in this pellet^38^, this pellet was referred to as the exosome pool or P100 pellet, with the resulting supernatant indicated as the S100 sup.

**Figure 1:**
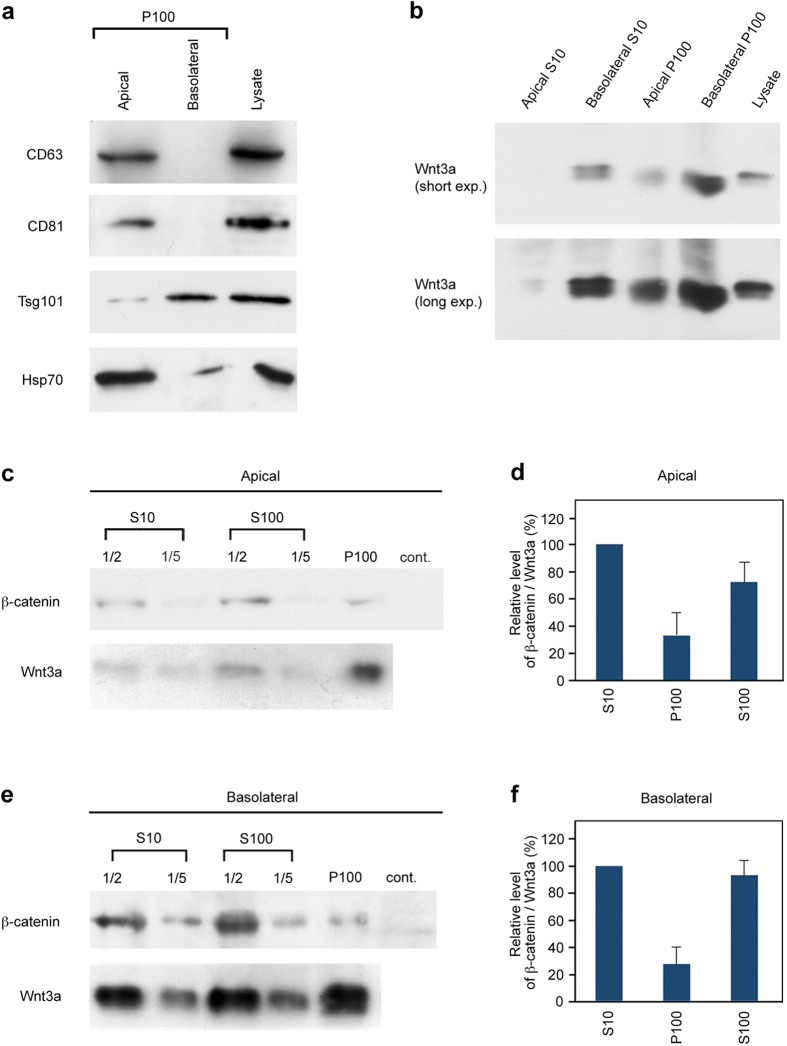
Wnt3a proteins were recovered into the exosome pool, P100 pellet, prepared from conditioned medium at the apical or basolateral side of MDCK cells. (**a**) Western blot analysis to examine relative amounts of exosome marker proteins, CD63, CD81, Tsg101, and Hsp70, recovered in P100 pellets of apical or basolateral medium of Wnt3a-expressing MDCK (MDCK-3a) cells. Equal amounts of P100 samples prepared from apical or basolateral medium were subjected to Western blotting. As standards, small amounts of cell lysates were also loaded. (**b**) The amounts of Wnt3a in S10 sup and P100 pellet from apical or basolateral medium of MDCK-3a cells, as well as the amount in the cell lysate, were analyzed by Western blotting. To compare the amounts of Wnt3a proteins between apical and basolateral media, an equal volume of S10 (10 μl each for apical or basolateral S10 pool) or equal amount of P100 (1/40 of P100 pellets prepared from 400 ml of apical or basolateral media) samples was loaded. In all MDCK cultures in this study, the same volume of medium was added to both apical and basolateral chambers. (**c–f**) Western blotting analysis for detection of activity and amount of Wnt proteins recovered in S10 sup, S100 sup or P100 pellet prepared from either the apical (**c**) or basolateral side (**e**) of MDCK cells. For monitoring of the Wnt3a activity, the amount of stabilized β-catenin induced by the addition of S10 sup, S100 sup or P100 pellet was quantified in L cells, in which β-catenin is undetectable without the addition of Wnt proteins (control; **c,e**). In parallel, the amount of Wnt3a used in this activity assay was analyzed (**c,e**). The amounts of β-catenin and Wnt3a in S10, S100 and P100, shown in (**c**) or (**e**), were quantified using Image J software. The ratios of β-catenin to Wnt3a level are indicated in numerical values relative to that in S10, which was set as 100 (**d,f**). The results shown are the mean ± S.D. from 3 independent experiments. Full-length blots are presented in Supplemental Fig. S4.

Several proteins that have been described to be abundant in exosomes were differently recovered in P100 pellets prepared from conditioned medium taken from the apical and basolateral sides of parental MDCK cells (Supplemental Fig. S2) and MDCK-3a cells (Fig. 1). In these cell populations, Tsg101 was mainly detected in the P100 pellet obtained from the basolateral side whereas CD63, CD81, and Hsp70 were preferentially recovered in that from the apical side (Fig. 1a, Supplemental Fig. S2a), suggesting that different populations of the exosomes may have been selectively secreted in a cell polarity-specific manner.

To examine this possibility more precisely, we further fractionated P100 by using continuous sucrose-gradient ultracentrifugation. Since previous studies showed that heterogeneous populations of exosomes can be separated under a certain condition of sucrose-gradient ultracentrifugation, we fractionated P100 under a condition almost identical to that described previously^11^. We loaded apical or basolateral P100 on the bottom, not on the top, of the sucrose gradient and then performed ultracentrifugation for 18 hrs. Under almost same condition, that is, centrifugation at 100,000 × g for 16 hrs, Willms *et al*. recovered exosomes in 2 populations, one corresponding to the typical density range of exosomes (1.12–1.19 g/ml) and the other showing a higher density^11^. Although these two populations are distinct after 16 hrs of centrifugation, the higher density population then becomes to equilibrate at the typical exosome density (1.12–1.19 g/ml) after 72 hrs of centrifugation. Thus, the higher density population appears to be a subpopulation of exosomes displaying delayed floatation behavior^11^.

**Figure 2:**
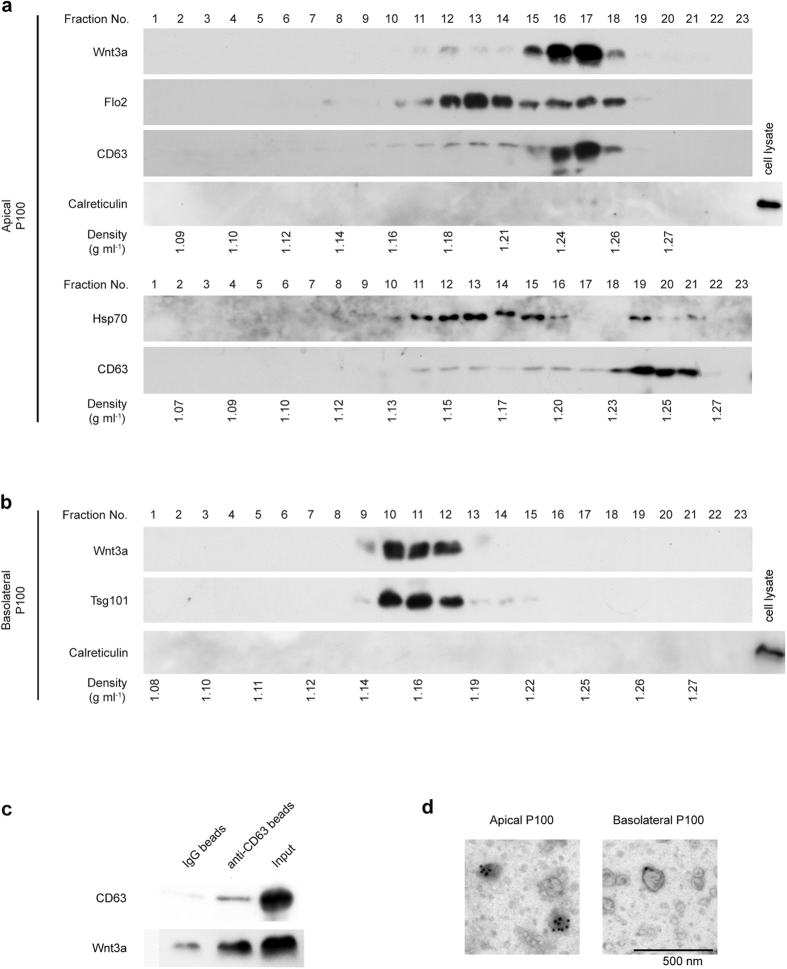
Characterization of Wnt3a-containing vesicles secreted from apical or basolateral side of MDCK cells. (**a,b**) The P100 pellet of either apical (**a**) or basolateral (**b**) conditioned medium from Wnt3a-expressing MDCK (MDCK-3a) cells was subjected to 0.25–2 M continuous sucrose density-gradient centrifugation. Equal volumes of the collected fractions were analyzed by Western blotting to detect Wnt3a and several markers of exosomes, including Flotillin2 (Flo2), CD63, and Tsg101. In addition, contamination of the ER in the apical sample was examined by use of Calreticulin antibody. Distribution of Hsp70 in apical P100 fractionations is indicated separately in comparison with that of CD63. The results are representative of 4 independent experiments. (**c**) Immunoprecipitation analysis to detect Wnt3a in CD63-containing vesicles. The P100 pellet prepared from apically secreted conditioned medium was incubated with anti-CD63-beads or IgG isotype-beads, and the complexes on the beads were recovered and examined with anti- Wnt3a and CD63 antibodies. (**d**) Immuno-electron microscopy of Wnt3a-containing exosomes released into apical and basolateral media. Vesicles stained with anti-Wnt3a antibody and gold particle-conjugated second antibody were observed by transmission electron microscopy. Full-length blots are presented in Supplemental Fig. S5.

In the fractions of the basolateral P100 pellet, Tsg101 was mainly recovered in fractions of density between 1.13 and 1.18 g/mL, where typical exosomes are detected (Fig. 2b and Supplemental Fig. S2c). In contrast, in the apical P100 fractions, CD63 was mainly recovered in higher density pools with a peak around 1.24 to 1.26 g/mL; although a small amount of this protein was also detected in lower density fractions (Fig. 2a and Supplemental Fig. S2b). Furthermore, Hsp70 in the apical P100 was separately recovered in fractions covering both the typical exosome pool and CD63-enriched high-density pool (Fig. 2a and Supplemental Fig. S2b). Flo2 in the apical P100 was also broadly distributed, but mostly recovered in fractions of density between 1.18 and 1.21 g/mL, close to, but slightly higher than the typical exosome density; although a small amount of Flo2 was also detected in the CD63-enriched higher density pool (Fig. 2b and Supplemental Fig. S2b). Thus, exosome markers recovered in the basolateral P100 were detected mainly in fractions with typical exosome density, whereas those in the apical one were recovered in 2 distinct pools that differed in their floatation behavior during sucrose-density gradient ultracentrifugation. These results suggest that exosomes, or exosomes-like vesicles, secreted from MDCK cells appeared to be heterogeneous in terms of secretory polarity, behavior during density ultracentrifugation, and composition of marker proteins. We refer to vesicles with densities close to those of the typical exosomes (1.10–1.20 g/mL)^39^ as conventional exosomes and those with higher density in the centrifugation condition of this study as high-density (HD) vesicle.

### Wnt3a was associated with different populations of vesicles secreted distinctly from the apical and the basolateral sides of MDCK cells

Western blot analysis of the P100 pellet prepared from MDCK-3a cells showed that small but detectable amounts of Wnt3a were reproducibly recovered from both the apical and basolateral pellets (Fig. 1b). However, we could not precisely estimate the recovery of Wnt3a proteins in these pellets, because it was difficult to dissolve the pellet completely. On the other hand, we confirmed that both apical and basolateral P100 pellets could significantly activate Wnt/β-catenin signaling by monitoring the β-catenin protein level in cadherin (−) L cells, in which β-catenin is undetectable without the addition of Wnt proteins. In terms of the specific activities (the ratios of β-catenin activation to Wnt3a expression), those of Wnt3a in the P100 pellets were lower than the average of the specific activity in the culture supernatant (Fig. 1c–f). The specific activity in the apical conditioned medium was almost similar to that in the basolateral one (Supplemental Fig. S1d,e), and also we could not find significant difference in the specific activity between apical and basolateral P100 pellets (Fig. 1d,f).

Analysis with sucrose-gradient ultracentrifugation showed that basolaterally released Wnt3a proteins in the P100 pellet were distributed in fractions of density between 1.14 and 1.18 g/mL (Fractions 9~12, Fig. 2b), where Tsg101 was detected, suggesting that a certain amount of Wnt3a could be secreted via conventional exosomes from the basolateral side of MDCK-3a cells. On the other hand, Wnt3a from the apical surface was detected only in fractions with a density corresponding to that of the HD vesicles (1.22–1.26 g/mL; Fractions 15–18 Fig. 2a). This form of Wnt3a was co-fractionated with CD63 (Fig. 2a). Calreticulin, which is an ER marker, was not detected in the Wnt3a-positive pools prepared from either basolateral or apical P100, suggesting that these 2 forms of Wnt3a were not associated with ER fragments released by cell death (Fig. 2a,b). These results suggest that Wnt3a was secreted in association with different populations of vesicles depending on the cell polarity.

Since most of the apically secreted Wnt3a recovered in the P100 pellet was co-fractionated with CD63, we further examined whether Wnt3a and CD63 actually co-existed on the same particle, or alternatively, whether some structure bearing these 2 molecules had a similar density. To differentiate between these possibilities, we used CD63-antibody-coated aldehyde-sulfate latex beads to capture CD63-containg structures, as described earlier^40^, and examined the co-existence of Wnt3a by immunoprecipitation and Western blotting. Compared to the IgG-coupled isotype control, CD63-coated beads trapped higher amounts of Wnt3a than did the control ones (Fig. 2c), indicating that Wnt3a and CD63 resided on the same particle. Immuno-electron microscopy indicated that Wnt3a proteins in the apical and basolateral P100 pellets were associated with particles around 100 nm in diameter, like exosomes (Fig. 2d). Thus, we concluded that apically and basolaterally secreted Wnt3a proteins were distinctly associated with different populations of exosomes, or exosome-like extracellular vesicles.

### Apically secreted Wnt11, like Wnt3a, was similarly associated with high density vesicles

**Figure 3:**
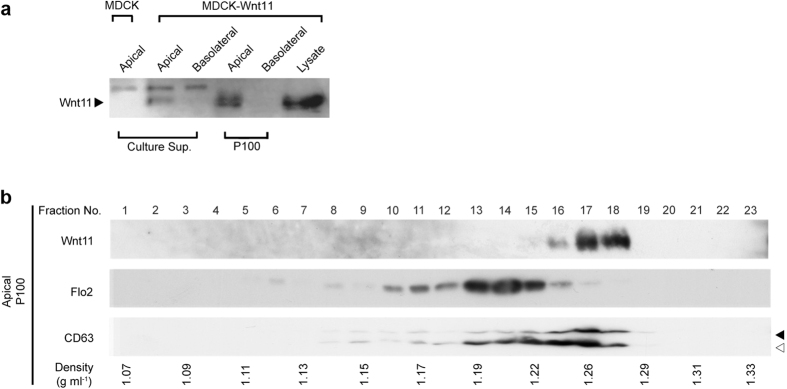
Wnt11 is associated with the apical CD63-containing vesicle. (**a**) Detection of Wnt11 (indicated by the closed arrow) in culture supernatant (Culture Sup.) and in P100 pellets. MDCK cells expressing Wnt11 were cultured in transwells, and soluble Wnt11 in the culture medium was concentrated by use of Blue Sepharose. Then, concentrated samples were suspended in distilled water; and P100 pellets were prepared as described for Wnt3a. Arrowheads indicate the position of Wnt11 protein. Upper bands shown in culture sup. are non-specifically cross-reactive. (**b**) Sucrose density-gradient centrifugation analysis of Wnt11. The P100 pellet prepared from the apical side of Wnt11-expressing MDCK cells was further fractionated by sucrose density-gradient as was shown in Fig. 2. Wnt11, Flo2, and CD63 were analyzed by Western blotting. (Full-length and smaller forms of CD63 are indicated by the closed and open arrow, respectively) Results shown are representative of 2 independent experiments. Full-length blots are presented in Supplemental Fig. S6.

There are 19 members of the Wnt protein family in humans and mice, but it has been barely examined whether these different Wnts are secreted in association with exosomes. Since Wnt11 is differently secreted than Wnt3a with respect to apico-basal polarity^34^, we next examined whether mouse Wnt11 was secreted in association with exosomes from MDCK cells or not. In agreement with previously published observations^34^, Wnt11 was mainly secreted into the apical compartment when Wnt11-expressing MDCK cells were cultured in transwells (Fig. 3a). Wnt11 was not detectable in the P100 pellet prepared from culture medium collected from the basolateral side. Furthermore, sucrose density-gradient centrifugation indicated that no association of Wnt11 with typical exosomes was detected when conditioned medium from either the apical or basolateral side was examined (Fig. 3b and data not shown). Instead, Wnt11 proteins were fractionated with a peak in the high-density fractions (1.24~1.28 g/mL); and they co-fractionated with CD63 (Fig. 3b). Thus, Wnt11 proteins were not secreted with conventional exosomes; but some of them seemed to be secreted from the apical side in a subpopulation of extracellular vesicles similar to the HD vesicles.

### Variation of Wnt3a-associated vesicle populations secreted from L and HEK293 cells

Wnt3a with a similar density was also detected when the P100 pellets prepared from L and HEK (Human embryonic kidney) 293 cells were analyzed. In the P100 pellet from the L cells, 2 different sub-populations based on density were detected (Supplemental Fig. S3a). One was distributed in fractions (Fractions 9~13) with a density between 1.15~1.18 g/mL; and the other, in those with one between 1.23~1.27 g/mL (Fractions 16~18). These 2 forms of Wnt well corresponded to the conventional exosomes and the HD vesicles, respectively (Supplemental Fig. S3a). On the other hand, most of the Wnt3a proteins in the P100 pellet of Wnt3a-expressing HEK293 cells was distributed into fractions (Fractions 17~19) with a density between 1.25~1.29 g/mL, similar to the HD vesicles (Supplemental Fig. S3b). These results indicate that Wnt3a was secreted via several different populations of vesicles depending on the cell type.

### The lipidation site was indispensable for Wnt3a loading onto the basolateral exosomes but not for association with the apical vesicles

**Figure 4:**
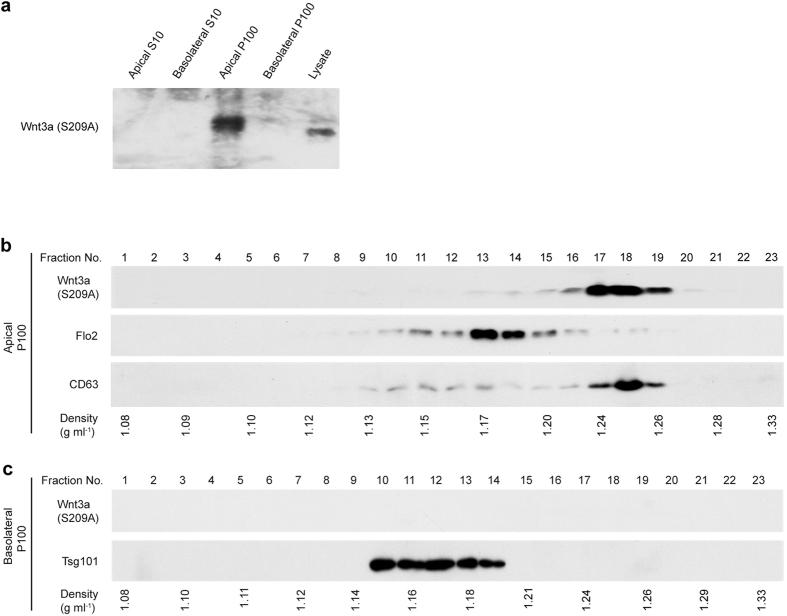
The lipidation site is essential for Wnt3a association with the basolateral exosome but not for association with the apically derived vesicle. (**a**) Western blot analysis of P100 pellets to detect recoveries of Wnt3a mutated at the lipidation site, Ser209, produced by MDCK cells. The amounts of Wnt3a in S10 sup and P100 pellet from conditioned medium, as well as the amount in the cell lysate, from MDCK cells expressing the lipidation site mutant Wnt3a (S209A), in which Ser209 was substituted to Ala, were analyzed by Western blotting. Comparative analysis with samples prepared from wild-type Wnt3a-expressing MDCK cells is shown in Fig. 1B. (**b,c**) Sucrose density-gradient centrifugation analysis of the lipidation site mutant. The P100 pellet from either the apical (**b**) or basolateral (**c**) side of MDCK cells expressing the S209A mutant of Wnt3a was subjected to continuous sucrose density-gradient as in Fig. 3. The amounts of Wnt3a, Flotillin2 (Flo2), CD63, and Tsg101 were analyzed by Western blotting. Results shown are representative of 3 independent experiments. Full-length blots are presented in Supplemental Fig. S7.

Wnt proteins generally require a specific lipidation for their secretion^41^; but in several exceptional cases, Wnt is secreted in a lipidation-independent manner^42^,^43^,^44^. To elucidate whether lipidation was required for secretion of the 2 distinct populations of Wnt3a-associated vesicles, we next examined whether lipidation-defective Wnt3a would be secreted or not in these different forms from MDCK cells. When a lipidation-defective Wnt3a mutant (Wnt3a (S209A)), in which serine 209 was mutated to alanine^41^, was expressed in MDCK cells, secretion of this mutant Wnt3a into either the apical or basolateral culture medium was barely detected in contrast to the wild-type Wnt3a (Figs 1b and 4a, data not shown). Analysis by sucrose-gradient ultracentrifugation indicated that the mutation at S209 also eliminated exosome release of Wnt3a from the basolateral side (Fig. 4c). In contrast, comparable expression of Wnt3a was still observed in the apical P100 pellet (Fig. 4a). Further examination by sucrose-gradient ultracentrifugation showed that Wnt3a was present in fractions with densities of 1.24~1.26 g/mL, similar to the case for wild-type Wnt3a. In addition, CD63 also co-fractionated with apical P100 Wnt3a (Fig. 4b). These results thus indicated that specific lipidation was required for Wnt3a secretion via conventional exosomes from the basolateral side, but was dispensable for Wnt3a secretion associated with HD vesicles from the apical side.

## Discussion

Exosomes are considered as important vesicles for intercellular communication. They transport proteins, including cell signaling molecules and their receptors, as well as RNA, DNA, and lipids^1^,^2^. Accumulating evidence supports the heterogeneity of exosome populations, which differ in their biophysical properties and repertories of proteins and RNA^8^,^9^,^10^,^11^. Such a heterogeneity gives rise to further questions as to whether the release of distinct subpopulations of exosomes is regulated depending on differentiation or malignant status of cells as well as on cell polarity. Another important question is whether a particular cargo is selectively loaded onto a specific population of exosomes. In this study, we made 2 major findings regarding the heterogeneity of exosomes: One was that polarized epithelial cells actually released different populations of exosomes, or exosome-like vesicles, in a polarity-specific manner. The other was that Wnt, a cell signaling protein, was targeted to different populations of vesicles depending on the cell polarity. Furthermore, our results strongly suggest that a lipidation-dependent secretion machinery was specifically required for Wnt targeting to one subpopulation, but not to another, of vesicles. Based on these findings, we will now discuss the heterogeneity of exosomes and mechanisms for targeting Wnt cargo to different vesicles.

Previous studies indicate that sucrose-density ultracentrifugation can separate exosomes into different subpopulations. Under a condition where samples were loaded on the bottom, but not on the top, of a sucrose gradient and separated by 18 hrs of centrifugation at 100,000 xg, exosome markers were recovered into several distinct populations with different densities; one is similar to the typical density of exosomes (1.12–1.19 g/mL), and the other is higher than this density^8^,^11^. Of note, particles and materials detected in higher density pools equilibrate at the typical exosome density when centrifugation is performed for a longer period (62 hrs–72 hrs). These results indicate that cells release different subpopulations of exosomes showing different floatation behaviors. In this study, we first examined the heterogeneity of exosome populations released from polarized epithelial cells by performing sucrose-density gradient ultracentrifugation under the condition where distinct populations of the exosomes were able to be separated as described above. In this condition, we found that distinct populations of exosomes, or exosome-like vesicles, were selectively released depending on the apico-basal polarity. While basolaterally released exosomes were mostly recovered in fractions whose density was similar to the typical exosome density, apically secreted ones were fractionated into 2 populations showing either the typical exosome density or a higher density. Thus, it seemed plausible that different populations of extracellular vesicles could be detected when we carefully examined them by sucrose density-gradient centrifugation.

It has been proposed that epithelial cells secrete different populations of exosomes probably reflecting asymmetrical secretion along the apico-basal axis. Analysis of exosomal proteins secreted from organoids derived from a colon carcinoma cell line showed that molecules known to be apically or basolaterally trafficked in general show different degrees of abundance in these 2 distinct exosome populations^19^. On the other hand, in the present study, we directly compared exosomes, or exosome-like vesicles, secreted from apical and basolateral sides of polarized MDCK cells and observed an asymmetrical secretion of exosomal proteins. From the basolateral side, a Tsg101-enriched population with a density identical to that of typical exosomes was secreted (basolateral conventional exosome). On the other hand, 2 distinct populations of vesicles were detected in the conditioned medium collected from the apical side, i.e., a CD63-enriched high-density subpopulation (apical HD vesicle) and Flo2-enriched one with a typical exosome density (apical conventional exosome). Thus, while exosomes with a typical density were secreted from both apical and basolateral sides, those with a higher density, which probably displayed delayed floatation behavior, were specifically secreted from the apical side. As far as we know, this is the first direct indication that polarized epithelial cells actually release different populations of exosomes, or exosome-like vesicles, in a polarity-dependent manner.

In this study, we found that Wnt proteins were secreted in associated with 2 distinct types of extracellar vesicles: Wnt3a, as well as Wnt11, were secreted via high-density vesicles from the apical side, whereas Wnt3a was secreted via conventional exosomes from the basolateral side. The difference in physical property and protein compositions, as well as polarity in secretion, strongly suggest that the machineries involved in these 2 secretion processes appear to be different. Interestingly, studies on MVB sorting showed that distinct populations of intraluminal vesicles are formed in MVBs by both ESCRT-dependent and independent mechanisms 13-15 (upper). In this study, we showed that Tsg101, a member of the ESCRT complex, was specifically associated with the basolaterally, but not apically, secreted population of vesicles (Fig. 2b). Thus, we may speculate that the formation of a basolaterally secreted population of Wnt-containing exosomes, which exhibit conventional exosome behavior in the ultracentrifugation, relies on the ESCRT-dependent sorting machinery to the MVB, although further analysis is required to determine their correlation.

This study also indicated that lipidation-dependency differs between these 2 secretion processes. In many cases of Wnt secretion, they require specific lipidation with mono-unsaturated fatty acid at an evolutionally conserved serine (S209 in mouse Wnt3a) by the acyltransferase known as Porcupine in the ER^41^. Upon lipidation, Wnt proteins become associated with a Wnt carrier protein called Wntless/Evi/Sprinter and are then escorted to the plasma membrane^45^,^46^,^47^,^48^. However, it was also shown that this secretion machinery is not required for Wnt secretion in some exceptional cases^44^. In this study, we showed that this machinery was specifically required for basolateral secretion, but not for apical secretion, of vesicle-mediated Wnt secretion. This result further supports an idea that these 2 different populations of Wnt-containing vesicles were distinctly formed. Interestingly, several studies indicated that exosomes carrying Wntless/Evi/Sprinter (Wls) are released from cells^23^,^26^. For instance, at the Drosophila larval neuromuscular junction, Wls-containing exosomes are released from presynaptic neurons. This release is required for the secretion of Wg, a Drosophila ortholog of Wnt1, indicating that a secreted Wnt is transported across synapses via Wls-associated exosomes^23^,^25^. Since the lipidation is essential for physical interaction of Wnt with Wls^49^, the lipidation-dependency in basolateral secretion of Wnt-containing exosomes may suggest that Wnt is loaded onto basolateral exosomes via Wls, as proposed previously in synaptic transport of Wg. In other words, we can also say that the lipidation-independent loading of Wnt onto apical vesicles appears to be an unrevealed mechanism for Wnt secretion.

Finally, we should note that a significant amount of Wnt proteins secreted from the apical or the basolateral side of Wnt3a-expressing MDCK cells showed a higher density than those of conventional exosomes and HD vesicles, as they settled down to the bottom of the tube under sucrose density-gradient centrifugation. Since almost all free proteins and protein complexes settle down to the bottom under the same conditions of centrifugation, we speculate that a significantly abundant pool of Wnt proteins was also secreted as free proteins. Therefore, to gain our insight into polarity-dependent secretion of Wnt, it is also important to reveal the higher order structure and composition of this abundant pool of high density Wnt proteins, in addition to the Wnt proteins associated with exosomes.

## Methods

### Cell culture and transfection

MDCK II, HEK293, and L cells, all of which were kindly provided by Dr. M. Takeichi, were maintained in Dulbecco’s modified Eagle’s medium (DMEM) or in a 1:1 mixture of DMEM and Ham’s F-12 medium supplemented with 8% fetal calf serum (FCS) and antibiotics. Polarized MDCK II cell cultures were established by plating the cells at 2 × 10^6^ cells in 100-mm Transwell filter chambers (Corning), and the cells were incubated for 7 days with a daily change of fresh culture medium. The integrity of the monolayer was verified by measuring the Trans-epithelial Electrical Resistance with a volt-ohm meter (Millipore). MDCK II and HEK293 cells stably expressing wild-type mouse Wnt3a, mutant Wnt3a (Wnt3a (S209A)) or wild-type mouse Wnt11 were established by transfection with wild-type Wnt3a, Wnt11 or Wnt3a (S209A) plasmid as indicated previously^50^. In these cells, Wnt genes were expressed under the control of the CMV promoter. Cells were selected and maintained in culture medium containing 400 μg/mL and 200 μg/mL G418, respectively. L cells stably expressing wild-type Wnt3a were established as previously described^41^. The supernatant from cultures of Wnt3a- or Wnt11-producing cells was prepared as previously described^50^.

### Antibodies and Western Blotting

Monoclonal anti-Wnt-3a and anti-β-catenin were described previously^41^. Rabbit polyclonal antibody against Wnt11 (ab31962) and chicken polyclonal antibody against Calreticulin (ab14234) were purchased from Abcam. Mouse anti-Tsg101 (612697), anti-CD81 (555675), and anti-Flotillin-2 (610383) antibodies were purchased from BD Transduction Laboratories. Rabbit polyclonal anti-CD63 antibody (orb11597) was obtained from Biorbyt. Western blotting was performed according to a standard protocol. Mouse anti-Hsp70 (H5147) antibody was purchased from Sigma.

### Isolation of P100 pellet

The P100 pellet was isolated according to the procedure described previously^38^. Cells were cultured in culture dishes or transwells; and after having reached confluence, they were washed twice with PBS and then cultured in DMEM supplemented with 8% exosome-depleted FCS. After 48 h (Wnt3a-expressing L or HEK293 cell) or 24 h (Wnt3a-expressinbg MDCK cell) of incubation, the conditioned medium was collected and centrifuged at 2000 × g for 10 min at 4 °C to remove dead cells and then re-centrifuged at 10,000 × g for 30 min at 4 °C to remove cell debris. The supernatant collected at this step was referred to as S10. This S10 supernatant was collected and then centrifuged at 100,000 × g for 90 min at 4 °C to pellet the exosomes or vesicles that sedimented at this speed. The pellet, referred to as the P100 pellet, was washed once in a large volume of PBS and re-suspended in PBS or 20 mM HEPES for further analysis.

### Sucrose density-gradient ultracentrifugation

A linear sucrose gradient, 0.25–2.0 M sucrose in 20 mM HEPES at pH 7.4, as described previously^38^, was prepared with a Gradient Master (BIOCOMP Model 107ip). Freshly prepared P100 pellet from either 400 mL of apical or basolateral culture medium was re-suspended in 1.1 mL of 2.5 M sucrose/HEPES solution and loaded onto the bottom of a sucrose gradient with a syringe. The gradient was then centrifuged at 100,000 × g for 18 h at 4 °C in a SW41 Ti rotor (Beckman Coulter). Fractions of 0.5 mL each were collected from the top of the centrifuge tube by using a Piston Gradient Fractionator (BIOCOMP). The density of individual fractions was calculated by use of a refractometer operated at 25 °C. The proteins were precipitated with trichloroacetic acid.

### Wnt3a activity assay

L cells (1.5 × 10^5^ cells) were introduced into 24-well culture plates on the day before the activity assay. After removal of the culture medium, 500 μL of control or Wnt3a-containing conditioned medium (S10, S100) or P100 pellet prepared from either 400 ml of apical or basolateral conditioned medium was dissolved in a final volume of 500 μL exosome-free DH10 medium and was added to the cells, which were then further incubated for 16 h. The cells were lysed in 50 μL of SDS-PAGE buffer and subjected to Western blotting to detect β-catenin protein, an indicator of Wnt3a activity.

### Immuno-precipitation of exosomes

For immunoprecipitation of exosomes, 4-μm-diameter aldehyde/sulfate latex beads (Molecular Probes) were incubated with anti-CD63 or goat IgG isotype control (Gene Tex) under gentle agitation at room temperature overnight, as previously described^40^. The P100 pellet isolated from apical MDCK conditioned medium was incubated with 1 × 10^5^ anti-CD63 or isotype control conjugated beads in 300 μL wash buffer (PBS containing 2% exosome-depleted FCS) overnight at 4 °C under gentle agitation. The reaction was then blocked by incubation with 300 μL of 200 mM glycine for 30 min on ice. Exosome-conjugated beads were sequentially washed 3 times with wash buffer, dissolved in 50 μL of SDS-PAGE buffer, and subjected to Western blotting for detection of Wnt3a and CD63.

### Immuno-electron microscopy

PBS-suspended P100 pellets prepared from apical or basolateral medium were incubated with purified rabbit anti-mouse Wnt3a polyclonal antibody and then with a second antibody coupled to gold particles. Subsequently, the samples were adsorbed onto carbon-coated electron microscopy grids. Grids were observed with a JEM-1010 transmission electron microscope operated at 80 kV.

## Additional Information

**How to cite this article**: Chen, Q. *et al*. Different populations of Wnt-containing vesicles are individually released from polarized epithelial cells. *Sci. Rep.* **6**, 35562; doi: 10.1038/srep35562 (2016).

## Supplementary Material

Supplementary Information

## References

[b1] EL Andaloussi S., Mager I., Breakefield X. O. & Wood M. J. Extracellular vesicles: biology and emerging therapeutic opportunities. Nat. Rev. Drug. Discov. 12, 347–357 (2013). 23584393 10.1038/nrd3978

[b2] Ellis T. N. & Kuehn M. J. Virulence and immunomodulatory roles of bacterial outer membrane vesicles. Microbiol. Mol. Biol. Rev. 74, 81–94 (2010). 20197500 10.1128/MMBR.00031-09 PMC2832350

[b3] Raposo G. & Stoorvogel W. Extracellular vesicles: exosomes, microvesicles, and friends. J. Cell Biol. 200 373–383 (2013). 23420871 10.1083/jcb.201211138 PMC3575529

[b4] Lee Y., El Andaloussi S. & Wood M. J. Exosomes and microvesicles: extracellular vesicles for genetic information transfer and gene therapy. Hum. Mol. Genet. 21, 125–134 (2012). 10.1093/hmg/dds317 22872698

[b5] Colombo M., Raposo G. & Thery C. Biogenesis, secretion, and intercellular interactions of exosomes and other extracellular vesicles. Annu. Rev. Cell Dev. Biol. 30, 255–289 (2014). 25288114 10.1146/annurev-cellbio-101512-122326

[b6] Mathivanan S., Ji H. & Simpson R. J. Exosomes: Extracellular organelles important in intercellular communication. J. Proteomics 73, 1907–1920 (2010). 20601276 10.1016/j.jprot.2010.06.006

[b7] Raiborg C. & Stenmark H. The ESCRT machinery in endosomal sorting of ubiquitylated membrane proteins. Nature 458, 445–452 (2009). 19325624 10.1038/nature07961

[b8] Palma J. . MicroRNAs are exported from malignant cells in customized particles. Nucleic Acids Res. 40, 9125–9138 (2012). 22772984 10.1093/nar/gks656 PMC3467054

[b9] Aalberts M. . Identification of distinct populations of prostasomes that differentially express prostate stem cell antigen, annexin A1, and GLIPR2 in humans. Biol. Reprod. 86, 82 (2012). 22133690 10.1095/biolreprod.111.095760

[b10] Bobrie A., Colombo M., Krumeich S., Raposo G. & Thery C. Diverse subpopulations of vesicles secreted by different intracellular mechanisms are present in exosome preparations obtained by differential ultracentrifugation. J. Extracell. Vesicles 1, 18397 (2012). 10.3402/jev.v1i0.18397 PMC3760636 24009879

[b11] Willms E. . Cells release subpopulations of exosomes with distinct molecular and biological properties. Sci. Rep. 6, 22519 (2016). 26931825 10.1038/srep22519 PMC4773763

[b12] Baietti M. F. . Syndecan-syntenin-ALIX regulates the biogenesis of exosomes. Nat. Cell Biol. 14, 677–685 (2012). 22660413 10.1038/ncb2502

[b13] van Niel G. . The tetraspanin CD63 regulates ESCRT-independent and -dependent endosomal sorting during melanogenesis. Dev. Cell 21, 708–721 (2011). 21962903 10.1016/j.devcel.2011.08.019 PMC3199340

[b14] Trajkovic K. . Ceramide triggers budding of exosome vesicles into multivesicular endosomes. Science 319, 1244–1247 (2008). 18309083 10.1126/science.1153124

[b15] Edgar J. R., Eden E. R. & Futter C. E. Hrs- and CD63-dependent competing mechanisms make different sized endosomal intraluminal vesicles. Traffic 15, 197–211 (2014). 24279430 10.1111/tra.12139 PMC4253088

[b16] Bu H. F., Wang X., Tang Y., Koti V. & Tan X. D. Toll-like receptor 2-mediated peptidoglycan uptake by immature intestinal epithelial cells from apical side and exosome-associated transcellular transcytosis. J. Cell Physiol. 222, 658–668 (2010). 20020500 10.1002/jcp.21985 PMC4414048

[b17] Liegeois S., Benedetto A., Garnier J. M., Schwab Y. & Labouesse M. The V0-ATPase mediates apical secretion of exosomes containing Hedgehog-related proteins in *Caenorhabditis elegans*. J. Cell Biol. 173, 949–961 (2006). 16785323 10.1083/jcb.200511072 PMC2063919

[b18] Sreekumar P. G. . alphaB crystallin is apically secreted within exosomes by polarized human retinal pigment epithelium and provides neuroprotection to adjacent cells. PLoS One 5, e12578 (2010). 20949024 10.1371/journal.pone.0012578 PMC2951891

[b19] Tauro B. J. . Two distinct populations of exosomes are released from LIM1863 colon carcinoma cell-derived organoids. Mol. Cell. Proteomics 12, 587–598 (2013). 23230278 10.1074/mcp.M112.021303 PMC3591653

[b20] Clevers H. & Nusse R. Wnt/beta-catenin signaling and disease. Cell 149, 1192–1205 (2012). 22682243 10.1016/j.cell.2012.05.012

[b21] Clevers H., Loh K. M. & Nusse R. Stem cell signaling. An integral program for tissue renewal and regeneration: Wnt signaling and stem cell control. Science 346, 1248012 (2014). 25278615 10.1126/science.1248012

[b22] Willert K. & Nusse R. Wnt proteins. Cold Spring Harb. Perspect. Biol. 4(9), a007864 (2012). 10.1101/cshperspect.a007864 PMC3428774 22952392

[b23] Korkut C. . Trans-synaptic transmission of vesicular Wnt signals through Evi/Wntless. Cell 139, 393–404 (2009). 19837038 10.1016/j.cell.2009.07.051 PMC2785045

[b24] Gross J. C., Chaudhary V., Bartscherer K. & Boutros M. Active Wnt proteins are secreted on exosomes. Nat. Cell Biol. 14, 1036–1045 (2012). 22983114 10.1038/ncb2574

[b25] Koles K. . Mechanism of evenness interrupted (Evi)-exosome release at synaptic boutons. J. Biol. Chem. 287, 16820–16834 (2012). 22437826 10.1074/jbc.M112.342667 PMC3351299

[b26] Beckett K. . Drosophila S2 cells secrete wingless on exosome-like vesicles but the wingless gradient forms independently of exosomes. Traffic 14, 82–96 (2013). 23035643 10.1111/tra.12016 PMC4337976

[b27] Luga V. . Exosomes mediate stromal mobilization of autocrine Wnt-PCP signaling in breast cancer cell migration. Cell 151, 1542–1556 (2012). 23260141 10.1016/j.cell.2012.11.024

[b28] Kispert A., Vainio S., Shen L., Rowitch D. H. & McMahon A. P. Proteoglycans are required for maintenance of Wnt-11 expression in the ureter tips. Development 122, 3627–3637 (1996). 8951078 10.1242/dev.122.11.3627

[b29] Pfeiffer S., Ricardo S., Manneville J. B., Alexandre C. & Vincent J. P. Producing cells retain and recycle Wingless in Drosophila embryos. Curr. Biol. 12, 957–962 (2002). 12062063 10.1016/s0960-9822(02)00867-9

[b30] Simmonds A. J., dosSantos G., Livne-Bar I. & Krause H. M. Apical localization of wingless transcripts is required for wingless signaling. Cell 105, 197–207 (2001). 11336670 10.1016/s0092-8674(01)00311-7

[b31] Wilkie G. S. & Davis I. Drosophila wingless and pair-rule transcripts localize apically by dynein-mediated transport of RNA particles. Cell 105, 209–219 (2001). 11336671 10.1016/s0092-8674(01)00312-9

[b32] Strigini M. & Cohen S. M. Wingless gradient formation in the Drosophila wing. Curr. Biol. 10, 293–300 (2000). 10744972 10.1016/s0960-9822(00)00378-x

[b33] Yamazaki Y. . Godzilla-dependent transcytosis promotes Wingless signalling in Drosophila wing imaginal discs. Nat. Cell Biol. 18, 451–457 (2016). 26974662 10.1038/ncb3325 PMC4817240

[b34] Yamamoto H. . The apical and basolateral secretion of Wnt11 and Wnt3a in polarized epithelial cells is regulated by different mechanisms. J. Cell Sci. 126, 2931–2943 (2013). 23613470 10.1242/jcs.126052

[b35] Katanaev V. L. . Reggie-1/flotillin-2 promotes secretion of the long-range signalling forms of Wingless and Hedgehog in Drosophila. EMBO J. 27, 509–521 (2008). 18219274 10.1038/sj.emboj.7601981 PMC2219691

[b36] Solis G. P., Luchtenborg A. M. & Katanaev V. L. Wnt secretion and gradient formation. Int. J. Mol. Sci. 14, 5130–5145 (2013). 23455472 10.3390/ijms14035130 PMC3634490

[b37] Balcarova-Stander J., Pfeiffer S. E., Fuller S. D. & Simons K. Development of cell surface polarity in the epithelial Madin-Darby canine kidney (MDCK) cell line. EMBO J. 3, 2687–2694 (1984). 6391916 10.1002/j.1460-2075.1984.tb02194.x PMC557750

[b38] Thery C., Amigorena S., Raposo G. & Clayton A. Isolation and characterization of exosomes from cell culture supernatants and biological fluids. Curr. Protoc Cell Biol. Chapter 3, Unit 3, 22 (2006). 18228490 10.1002/0471143030.cb0322s30

[b39] Raposo G. . B lymphocytes secrete antigen-presenting vesicles. J Exp Med. 183, 1161–1172 (1996). 8642258 10.1084/jem.183.3.1161 PMC2192324

[b40] Lasser C., Eldh M. & Lotvall J. Isolation and characterization of RNA-containing exosomes. J. Vis. Exp. 59, e3037 (2012). 22257828 10.3791/3037 PMC3369768

[b41] Takada R. . Monounsaturated fatty acid modification of Wnt protein: its role in Wnt secretion. Dev. Cell 11, 791–801 (2006). 17141155 10.1016/j.devcel.2006.10.003

[b42] Ching W., Hang H. C. & Nusse R. Lipid-independent secretion of a Drosophila Wnt protein. J. Biol. Chem. 283, 17092–17098 (2008). 18430724 10.1074/jbc.M802059200 PMC2427328

[b43] Franch-Marro X., Wendler F., Griffith J., Maurice M. M. & Vincent J. P. *In vivo* role of lipid adducts on Wingless. J. Cell Sci. 121, 1587–1592 (2008). 18430784 10.1242/jcs.015958 PMC7611555

[b44] Chen Q., Takada R. & Takada S. Loss of Porcupine impairs convergent extension during gastrulation in zebrafish. J. Cell Sci. 125, 2224–2234 (2012). 22357957 10.1242/jcs.098368

[b45] Banziger C. . Wntless, a conserved membrane protein dedicated to the secretion of Wnt proteins from signaling cells. Cell 125, 509–522 (2006). 16678095 10.1016/j.cell.2006.02.049

[b46] Bartscherer K., Pelte N., Ingelfinger D. & Boutros M. Secretion of Wnt ligands requires Evi, a conserved transmembrane protein. Cell 125, 523–533 (2006). 16678096 10.1016/j.cell.2006.04.009

[b47] Fu J., Jiang M., Mirando A. J., Yu H. M. & Hsu W. Reciprocal regulation of Wnt and Gpr177/mouse Wntless is required for embryonic axis formation. Proc. Natl. Acad. Sci. USA 106, 18598–18603 (2009). 19841259 10.1073/pnas.0904894106 PMC2773984

[b48] Goodman R. M. . Sprinter: a novel transmembrane protein required for Wg secretion and signaling. Development 133, 4901–4911 (2006). 17108000 10.1242/dev.02674

[b49] Coombs G. S. . WLS-dependent secretion of WNT3A requires Ser209 acylation and vacuolar acidification. J. Cell Sci. 123, 3357–3367 (2010). 20826466 10.1242/jcs.072132 PMC2939803

[b50] Shibamoto S. . Cytoskeletal reorganization by soluble Wnt-3a protein signalling. Genes Cells 3, 659–670 (1998). 9893023 10.1046/j.1365-2443.1998.00221.x

