# Supplementary material for: Different populations of Wnt-containing vesicles are individually released from polarized epithelial cells
Source: Sci Rep. 2016 Oct 21;6:35562. doi: 10.1038/srep35562 (PMC5073244; doi:10.1038/srep35562)
Supplement: Supplementary Information [file srep35562-s1.pdf]

Supplemental Information

Different populations of Wnt-containing vesicles are individually released from polarized epithelial cells

Qiuhong Chen,[1,2] Ritsuko Takada,[1,2] Chiyo Noda,[1,2] Satoru Kobayashi,[1, 2, 3,4] & Shinji Takada[1, 2, 3,]

[1]*Okazaki Institute for Integrative Bioscience, National Institutes of Natural Sciences, Okazaki, Aichi 444-8787, Japan*

[2]*National Institute for Basic Biology, National Institutes of Natural Sciences, Okazaki, Aichi 444-8787, Japan*

[3]*The Graduate University for Advanced Studies (SOKENDAI), Okazaki, Aichi 444-8787, Japan*

[4]*Life Science Center, Tsukuba Advanced Research Alliance, University of Tsukuba, Tsukuba, Ibaraki 305-8577, Japan*

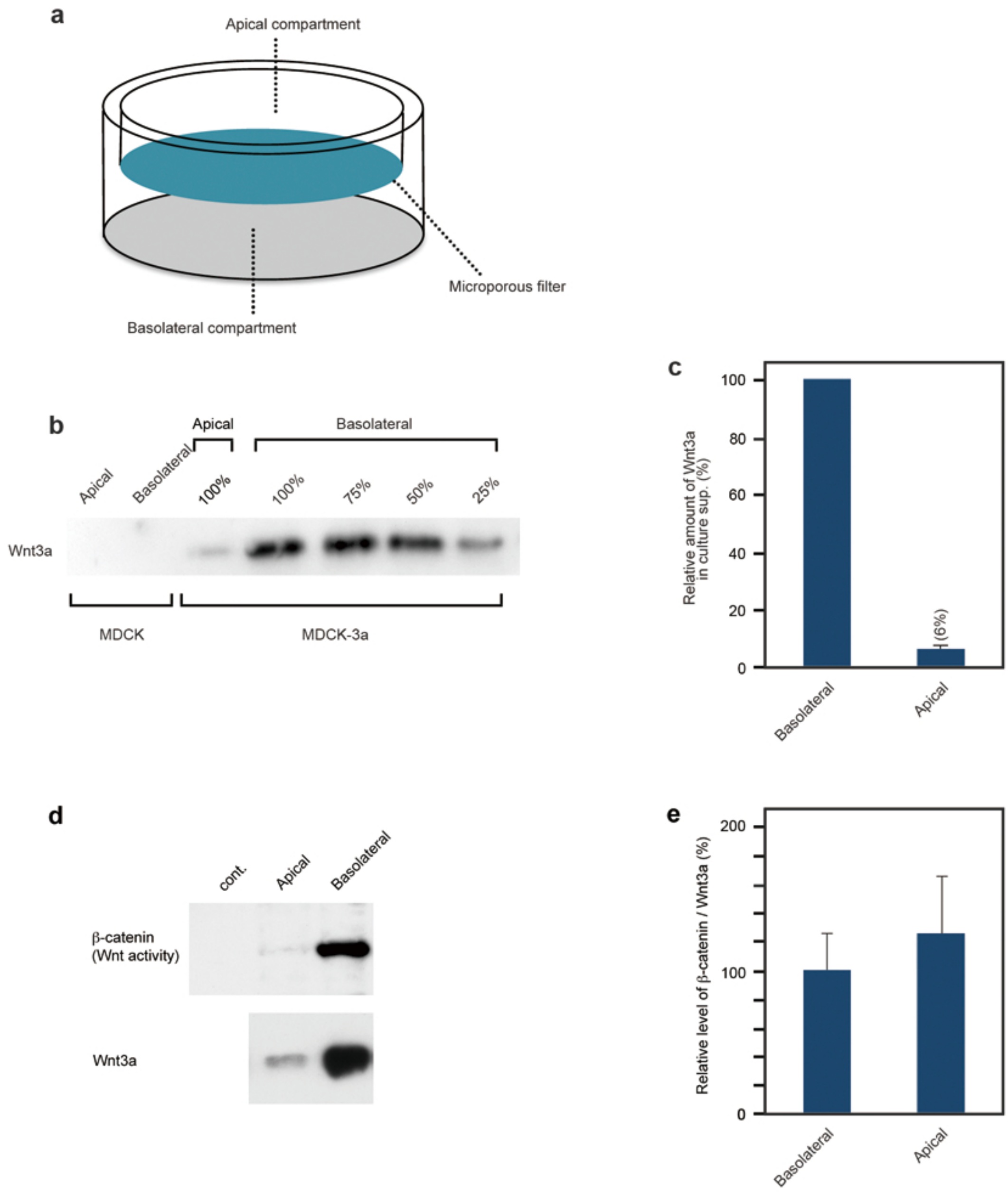

Supplemental Fig. S1. Wnt3a secretion from polarized MDCK cells.

(a) Schematic representation of a MDCK monolayer culture in a transwell. **(b)** Western blot analysis of Wnt3a secreted from either apical or basolateral side of Wnt3a-expressing MDCK cells. The same amount of conditioned medium prepared from both the apical and basolateral sides of MDCK and Wnt3a-expressing MDCK cells was loaded with a dilution series of basolateral conditioned medium from Wnt3a-expressing MDCK cells. **(c)** The percentage of

Wnt3a secreted from the apical side relative to that from the basolateral side (set as 100) is indicated. Results were obtained from 4 independent experiments and are shown as the mean ±S.D. **(d)** Western blotting analysis for detection of activity and amount of Wnt proteins secreted from either the apical or basolateral side of MDCK cells. For monitoring of the Wnt3a activity, the amount of stabilized β-catenin induced by the addition of the same volume of conditioned medium was quantified in L cells, in which β-catenin is undetectable without the addition of Wnt proteins. In parallel, the amount of Wnt3a used in this activity assay was analyzed. **(e)** The β-catenin level to Wnt3a proteins secreted from the apical side relative to that from the basolateral side (set as 100) is indicated. The amounts of β-catenin and Wnt3a were quantified using Image J software. The ratio of β-catenin to Wnt3a level is indicated as shown in Figure 1(d) and (f). The results shown are the mean ± S.D. from 3 independent experiments.

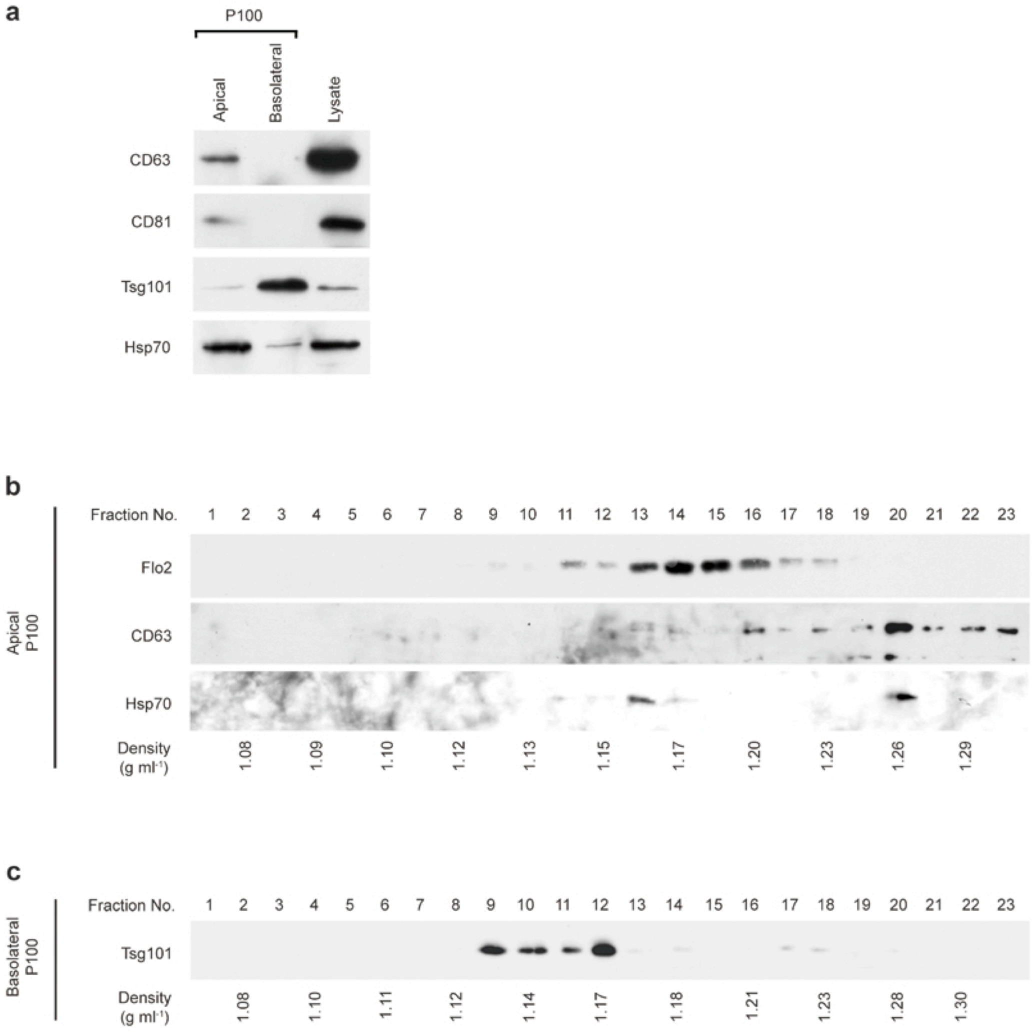

Supplemental Fig. S2. Distinct subpopulations of exosomes are released into apical and basolateral exosomes prepared from MDCK cells

(a) Western blotting analysis for detection of amounts of exosome marker proteins, CD63, CD81, Tsg101, and Hsp70, in the P100 pellet prepared either from either the apical or basolateral side of parental MDCK cells. Equal amounts of P100 samples prepared from apical or basolateral medium were subjected to Western blotting. As standards, small amounts of cell lysates were also loaded. **(b, c)** The P100 pellet of either apical **(b)** or basolateral **(c)** conditioned medium from MDCK cells was subjected to 0.25–2 M continuous sucrose density-gradient centrifugation. Equal volumes of the collected fractions were analyzed by Western blotting to detect several markers of the exosomes, including Flotillin2 (Flo2), CD63, Hsp70, and Tsg101.

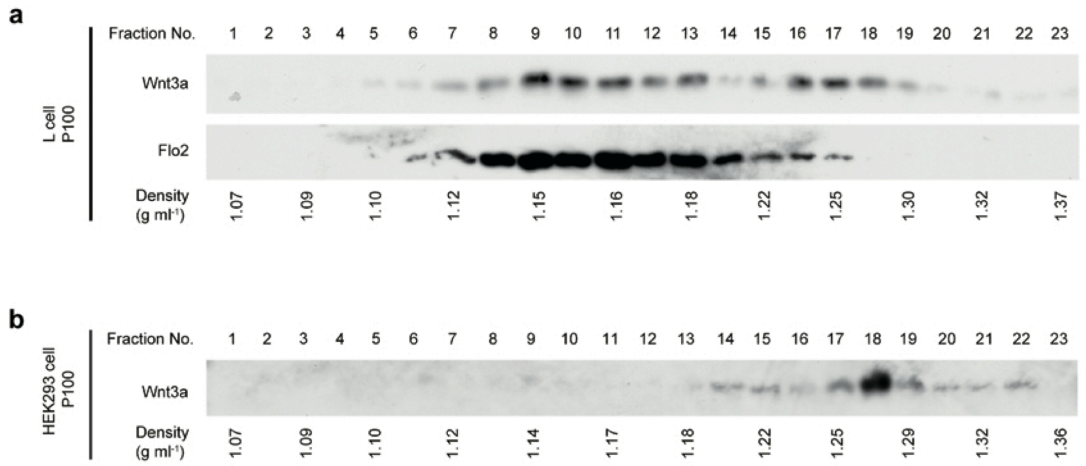

Supplemental Fig. S3. Analysis of Wnt3a in P100 pellets from L and HEK293 cells.

P100 pellets prepared from Wnt3a-expressing L **(a)** or HEK293 cells **(b)** were subjected to sucrose density-gradient centrifugation. The amounts of Wnt3a and Flotillin2 (Flo2) were analyzed by Western blotting.

For Figure 1-a

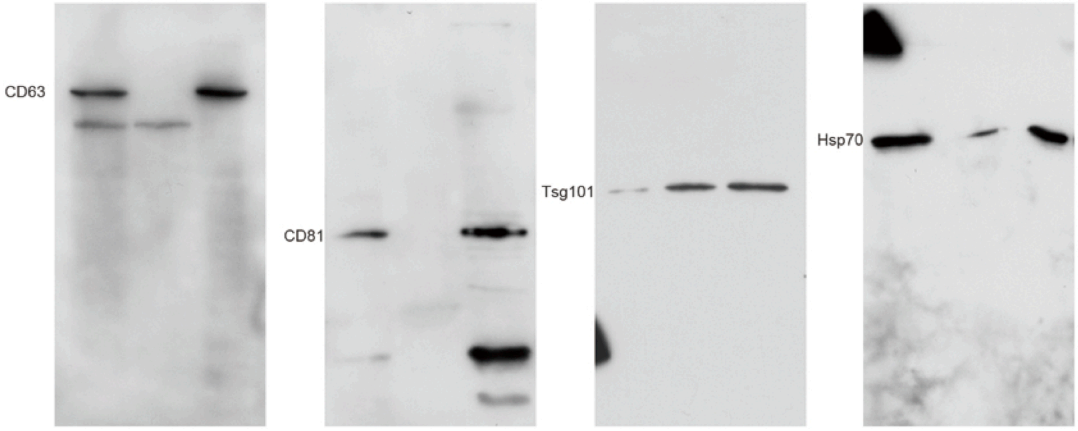

For Figure 1-b

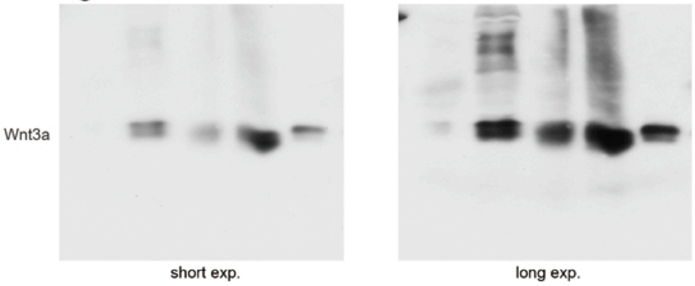

For Figure 1-c

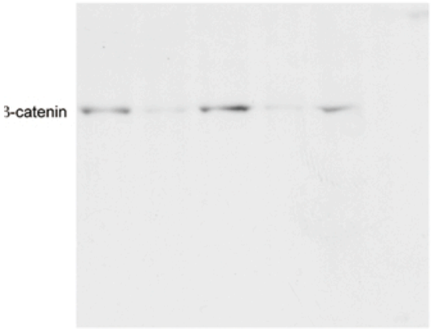

For Figure 1-e

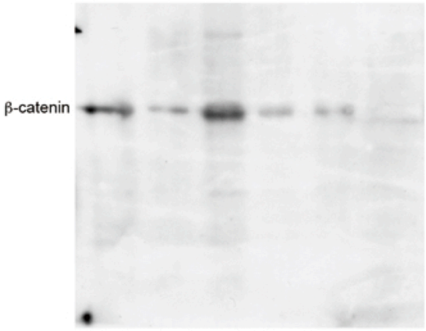

For Fig.1-c

For Fig.1-e

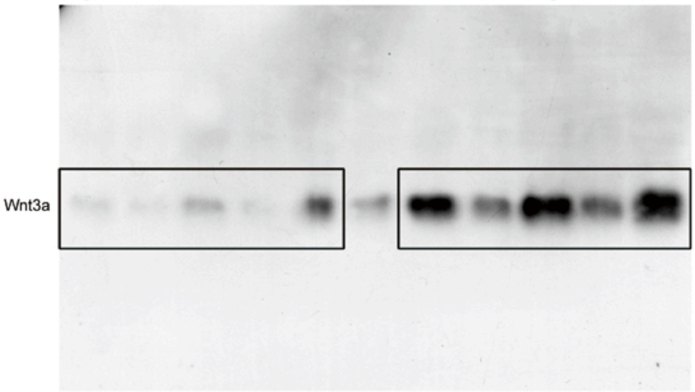

Supplemental Fig. S4. The original blots of Fig. 1 in the text.

For Figure 2-a

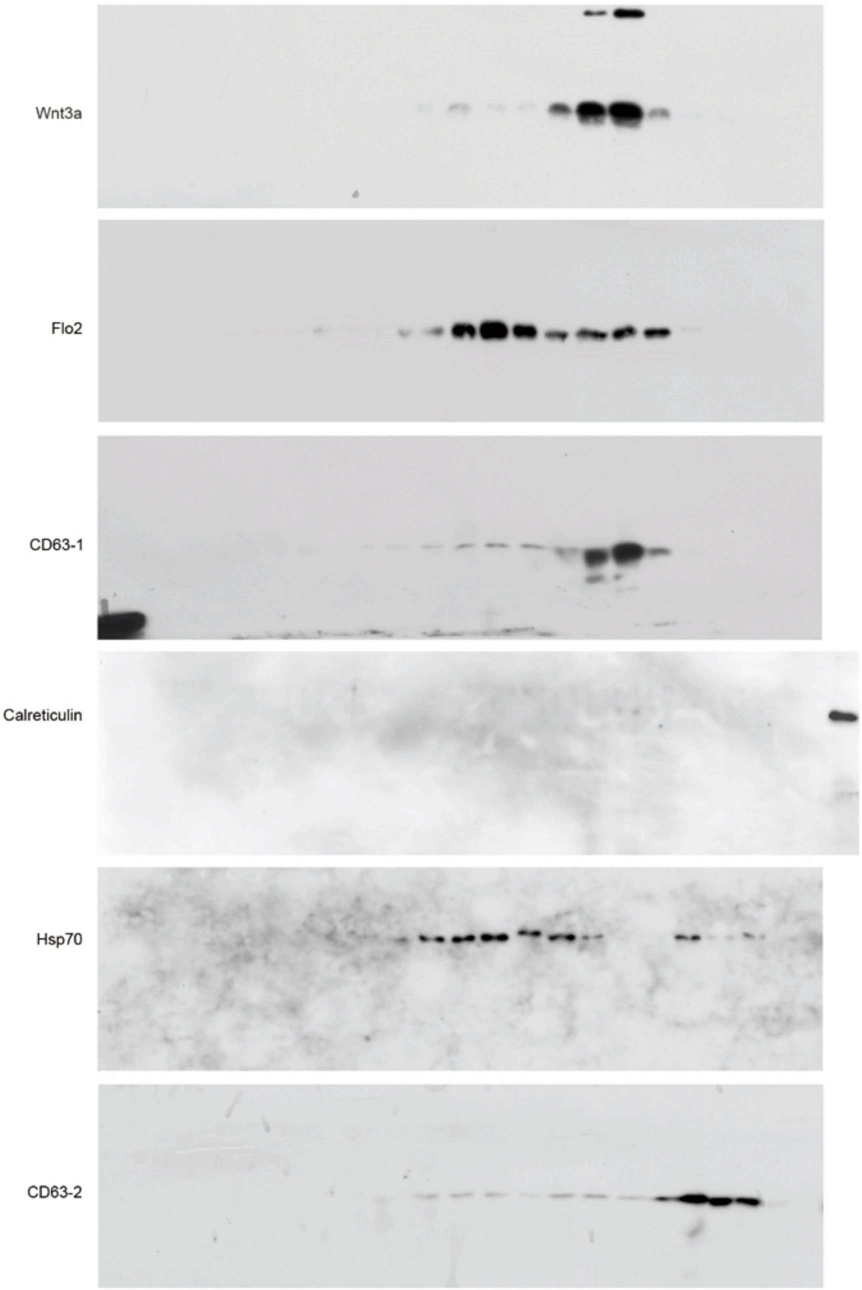

Supplemental Fig. S5-1. The original blots of Fig. 2 in the text.

For Figure 2-b

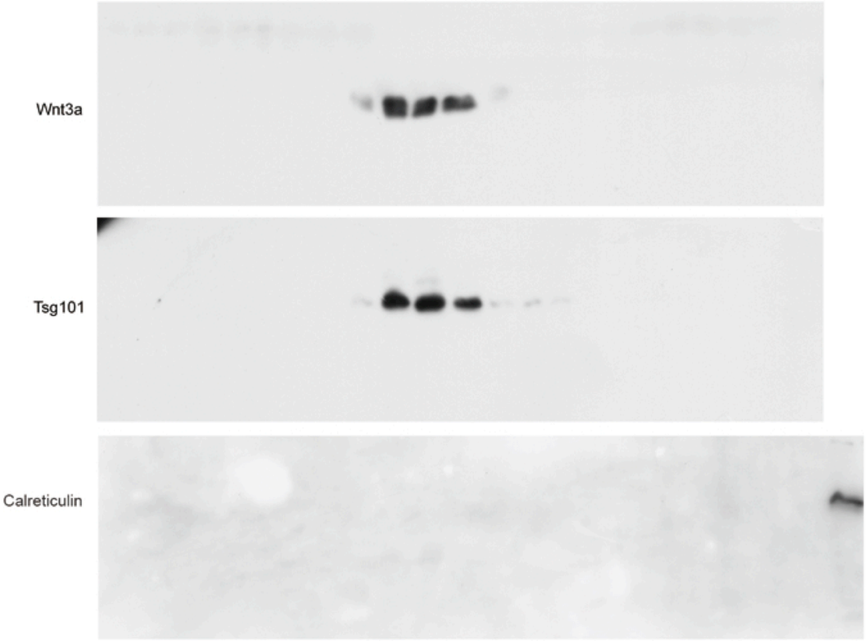

Figure 2-c

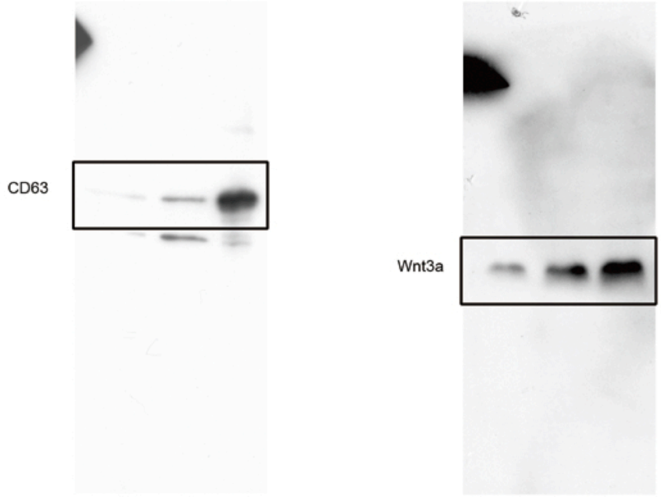

Supplemental Fig. S5-2. The original blots of Fig. 2 in the text.

For Figure 3-a

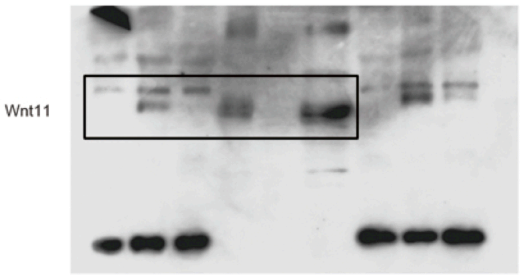

For Figure 3-b

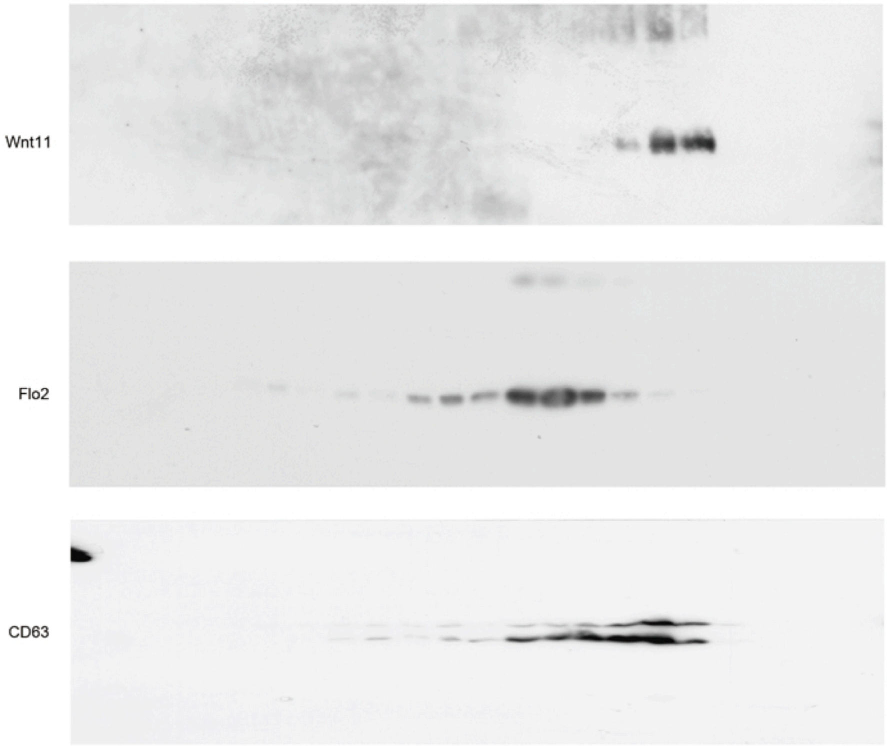

Supplemental Fig. S6. The original blots of Fig. 3 in the text.

For Figure 4-a

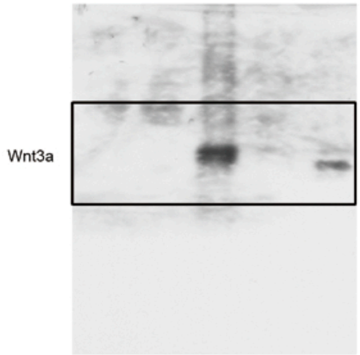

For Figure 4-b

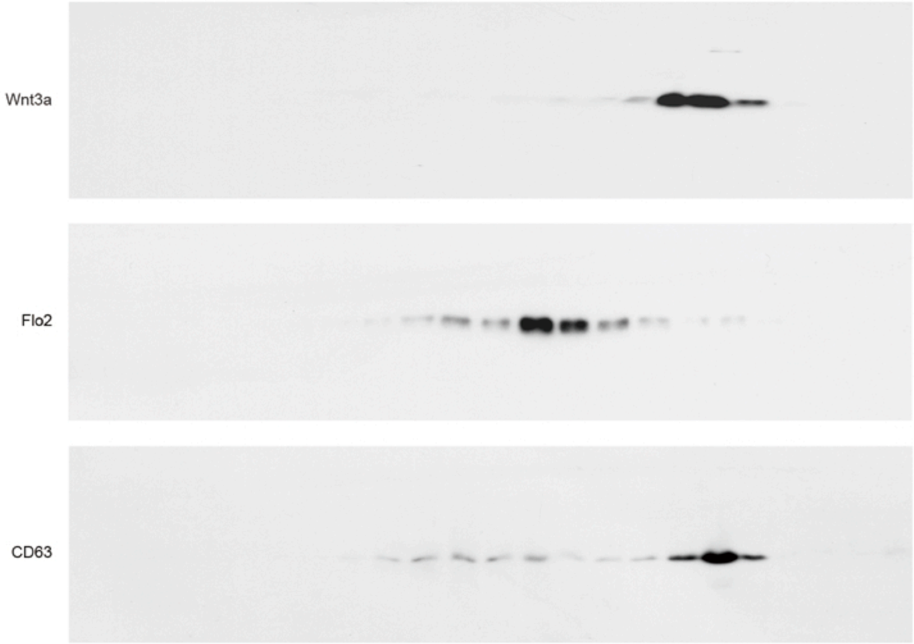

For Figure 4-c

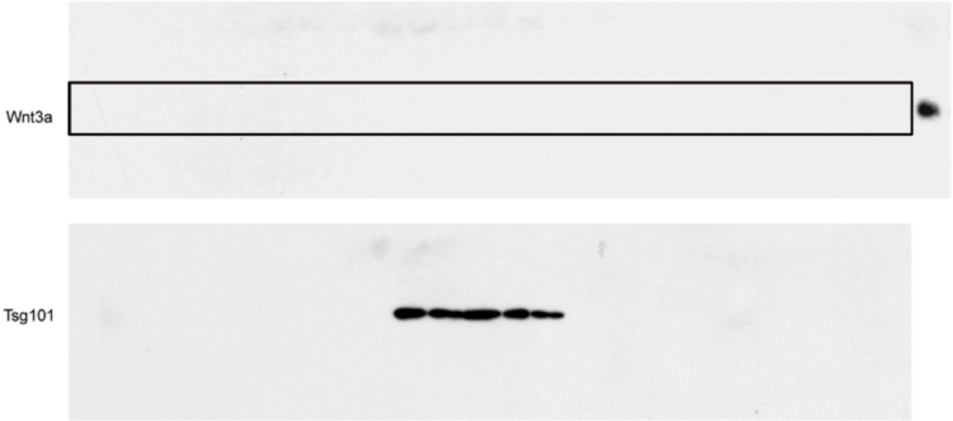

Supplemental Fig. S7. The original blots of Fig. 4 in the text.
